# Inhibition of selenoprotein synthesis is not the mechanism by which auranofin inhibits growth of *Clostridioides difficile*

**DOI:** 10.1038/s41598-023-36796-9

**Published:** 2023-09-07

**Authors:** Michael A. Johnstone, Matthew A. Holman, William T. Self

**Affiliations:** Burnett School of Biomedical Sciences, College of Medicine, University of Central Florida, 4110 Libra Drive, Orlando, FL 32816 USA

**Keywords:** Antimicrobials, Bacteriology

## Abstract

*Clostridioides difficile* infections (CDIs) are responsible for a significant number of antibiotic-associated diarrheal cases. The standard-of-care antibiotics for *C. difficile* are limited to fidaxomicin and vancomycin, with the recently obsolete metronidazole recommended if both are unavailable. No new antimicrobials have been approved for CDI since fidaxomicin in 2011, despite varying rates of treatment failure among all standard-of-care drugs. Drug repurposing is a rational strategy to generate new antimicrobials out of existing therapeutics approved for other indications. Auranofin is a gold-containing anti-rheumatic drug with antimicrobial activity against *C. difficile* and other microbes. In a previous report, our group hypothesized that inhibition of selenoprotein biosynthesis was auranofin’s primary mechanism of action against *C. difficile*. However, in this study, we discovered that *C. difficile* mutants lacking selenoproteins are still just as sensitive to auranofin as their respective wild-type strains. Moreover, we found that selenite supplementation dampens the activity of auranofin against *C. difficile* regardless of the presence of selenoproteins, suggesting that selenite’s neutralization of auranofin is not because of compensation for a chemically induced selenium deficiency. Our results clarify the findings of our original study and may aid drug repurposing efforts in discovering the compound’s true mechanism of action against *C. difficile*.

## Introduction

*Clostridioides difficile* (formerly *Clostridium difficile*) is a Gram-positive, endospore-forming strict anaerobe and the leading cause of antibiotic-associated diarrhea (~15–25% of cases)^1,2^. *C. difficile* infections (CDIs) typically occur in patients with gut dysbiosis and can lead to severe clinical complications such as pseudomembranous colitis and toxic megacolon^3^. During infection, *C. difficile* causes disease and induces inflammation by producing two large exotoxins—TcdA and TcdB—which damage the intestinal lining through the glucosylation of Rho-family GTPases in host epithelial cells^4^. According to a recent CDC report, CDIs were responsible for approximately 223,900 hospitalized patient cases and 12,800 deaths in 2017^5^. Moreover, CDIs have contributed to approximately $1 billion in U.S. healthcare costs^5^.

The standard-of-care antibiotics for treating CDI are fidaxomicin and vancomycin^6,7^. If neither drug is available, metronidazole is recommended as an alternative^6,7^, though this former first-line antibiotic is regarded as obsolete due to its high rates of treatment failure^8^. In fact, CDI recurrence occurs in ~15–30% of patients treated with metronidazole and vancomycin despite their effectiveness in inhibiting *C. difficile* growth^8,9^. On the other hand, fidaxomicin is a narrow-spectrum antimicrobial with greater potency and is typically associated with comparatively lower recurrence rates^10,11^, though treatment failure has also been reported^12^. Moreover, while not the primary issue encountered in CDI management, antimicrobial resistance is still a cause for concern as drug-resistant clinical isolates have been reported for all three antibiotics^13–16^. Overall, the current repertoire for treatment is quite limited, especially since fidaxomicin was the last CDI drug approved by the U.S. Food and Drug Administration (FDA) in 2011^17^. If no new alternatives are added to the current list of standard-of-care antibiotics, the rising rates of recurrence and antibiotic resistance could outpace efforts to keep CDI under reasonable control.

Auranofin is an FDA-approved anti-rheumatic gold (Au) compound that possesses antimicrobial activity against *C. difficile*^18,19^. Many reports have highlighted its inhibitory activity against *C. difficile* vegetative cells and sporulation, its ability to reduce toxin levels and protect Caco-2 cells against their lethal effects, and its efficacy in preventing CDI and disease recurrence in mouse and hamster models^20–24^. While the mechanism of action is still unknown, our group hypothesized that auranofin’s antimicrobial activity against *C. difficile* stemmed from its unique ability to halt the biosynthesis of selenoproteins (i.e., proteins containing the 21st amino acid selenocysteine)^19^. In *C. difficile*, the established selenoproteins are selenophosphate synthetase (SelD), D-proline reductase (Prd), and glycine reductase (Grd)^19,25^. SelD possesses the highly specific role of converting toxic selenide to selenophosphate, a selenium (Se) donor that is required for selenoprotein synthesis^26–28^. Prd and Grd are involved in Stickland metabolism, an important clostridial bioenergetics scheme centered on amino acid redox reactions^25,29^. Our group has previously shown via mass spectrometry and X-ray absorption spectroscopy that auranofin forms a Au-Se adduct with selenide in the culture medium; additionally, we have demonstrated via ^75^Se radiolabeling that auranofin inhibits uptake and incorporation of Se into selenoproteins in *C. difficile*^19^. Based on these data, we assumed that auranofin’s mechanism of action against *C. difficile* involved blocking Se transport through the formation of the Au-Se adduct, thereby crippling the production of crucial selenoproteins such as Prd and Grd^19^.

However, despite the perceived importance of selenoproteins in *C. difficile*, it is now known that they are not essential since *C. difficile selD* mutants derived from two different ribotypes are both clearly viable^28^. These findings prompted us to revisit our previous work on auranofin and determine if the compound’s activity against the pathogen is truly from its inhibition of Se metabolism^19^. Since that publication, the rapid advancement of genetic techniques to study clostridia has allowed for more precise investigations into the role of selenoproteins in *C. difficile*^28–32^. In this study, we determined the auranofin sensitivity of a panel of *C. difficile* mutants deficient in some or all selenoproteins in order to gain more insight on the compound’s mechanism of action.

## Results

### Wild-type *C. difficile* and mutants lacking selenoproteins exhibit similar sensitivity to auranofin

To determine if auranofin inhibits *C. difficile* by targeting Se metabolism, we evaluated the auranofin sensitivity of an array of *C. difficile* strains deficient in some or all selenoproteins (Table 1) using a modified version of the Clinical and Laboratory Standards Institute (CLSI) broth microdilution method^33^. While CLSI recommends the agar dilution method for antimicrobial susceptibility testing of anaerobes^33^, we chose broth microdilution because of its practicality and less cumbersome methodology. Moreover, broth microdilution has been reported to perform similarly to agar dilution in susceptibility tests of *C. difficile*^34,35^, though we are aware that others have observed substantial differences between both methods and argue against routine testing with broth microdilution^36,37^. Since our goal was to compare relative differences in minimum inhibitory concentrations (MICs) between strains rather than report standardized values that could be translated to the clinic, broth microdilution was therefore deemed appropriate for this study.

**Table 1:** Bacterial strains used in this study.

Bacterial strain	Description (relevant genotype)	Reference/source
R20291	Wild type, ribotype 027	^28^
KNM6	R20291 (Δ*selD*) CRISPR-Cas9 mutant	^28^
KNM9	KNM6 (Δ*selD*::*selD*^+^) CRISPR-Cas9 mutant	^31^
JIR8094	Wild type, ribotype 012, Erm^S^ derivative of strain 630	^29^
LB-CD4	JIR8094 (*prdB*::*ermB*) TargeTron mutant	^29^
LB-CD7	JIR8094 (*selD*::*ermB*) TargeTron mutant	^28^
LB-CD8	JIR8094 (*prdR*::*ermB*) TargeTron mutant	^29^
LB-CD12	JIR8094 (*grdA*::*ermB*) TargeTron mutant	^29^

**Figure 1:**
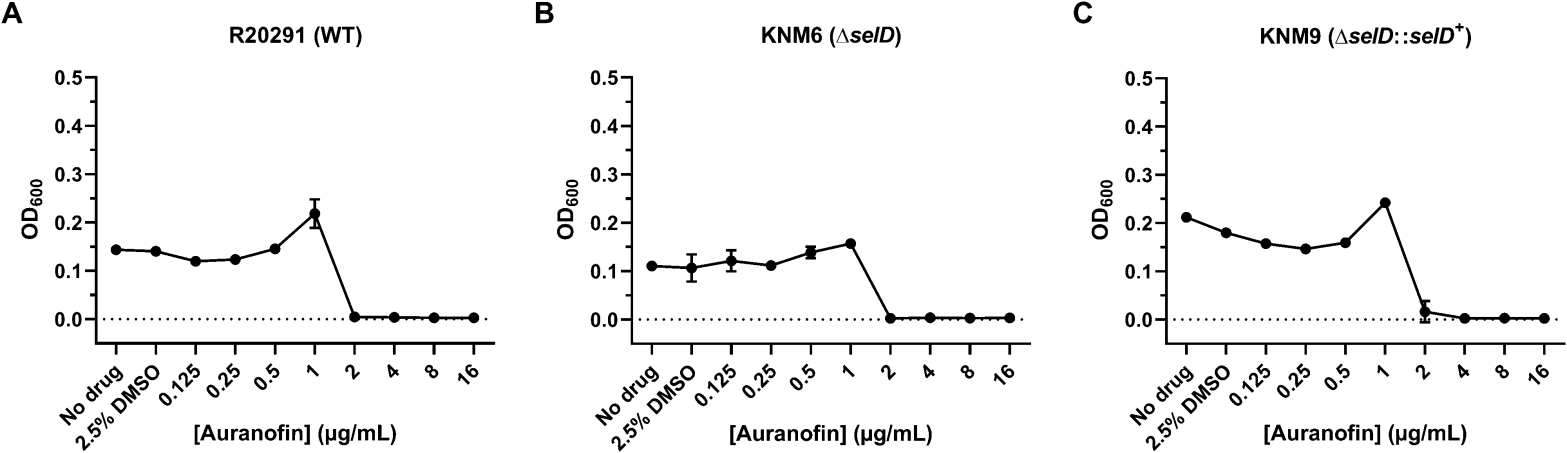
A *C. difficile* Δ*selD* mutant has the same sensitivity to auranofin as wild type. *C. difficile* strains (**A**) R20291, (**B**) KNM6, and (**C**) KNM9 were grown in BHIS broth augmented with 2.5% DMSO and varying concentrations of auranofin at 37 °C for 48 h. The OD_600_ of each culture was recorded at 48 h. The experiment was performed twice. Data points represent the means of triplicate cultures while error bars represent standard deviations.

**Figure 2:**
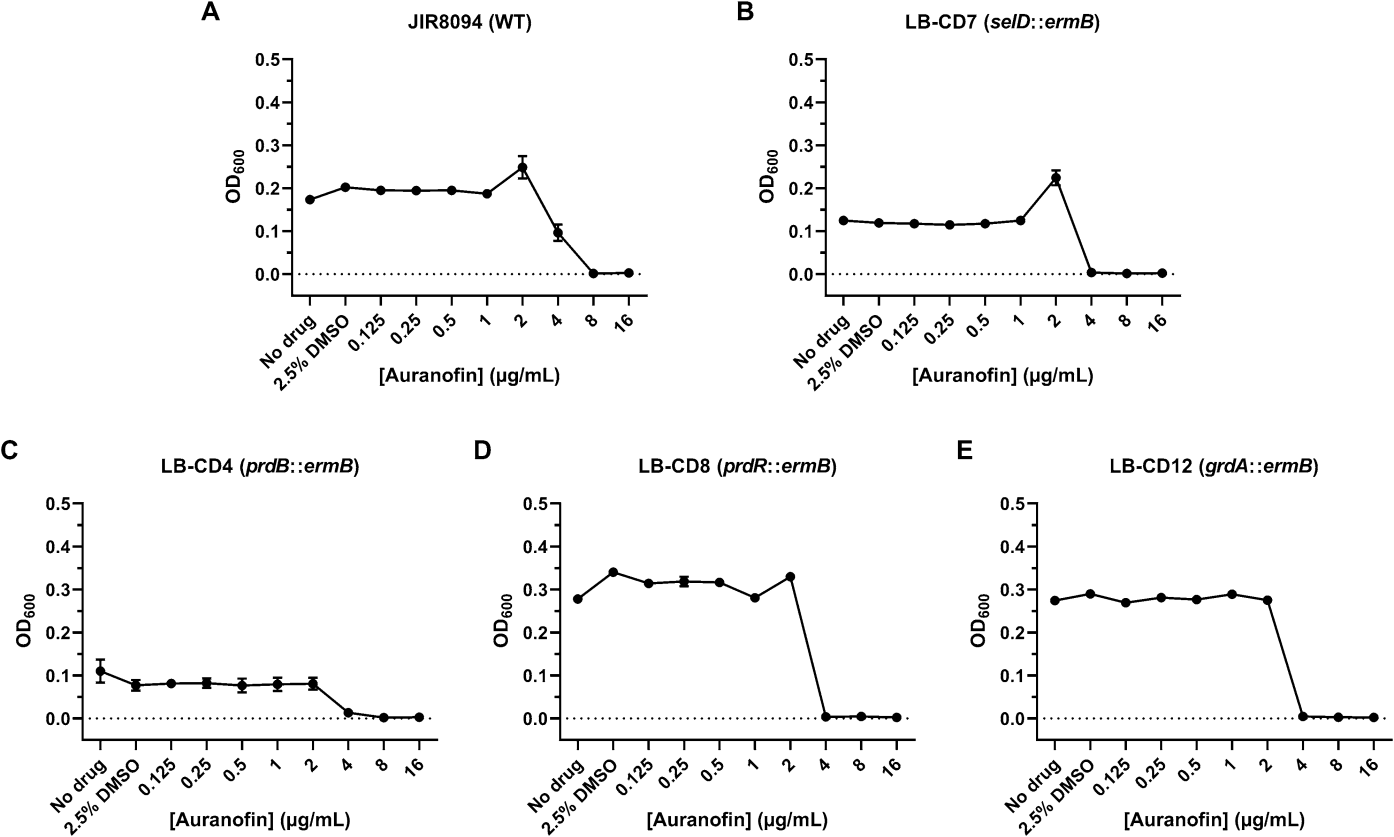
Mutations in selenophosphate synthetase, proline reductase, or glycine reductase do not confer resistance to auranofin. *C. difficile* strains (**A**) JIR8094, (**B**) LB-CD7, (**C**) LB-CD4, (**D**) LB-CD8, and (**E**) LB-CD12 were grown in BHIS broth augmented with 2.5% DMSO and varying concentrations of auranofin at 37 °C for 48 h. The OD_600_ of each culture was recorded at 48 h. The experiment was performed twice. Data points represent the means of triplicate cultures while error bars represent standard deviations.

Briefly, we cultured each strain in supplemented brain heart infusion (BHIS) broth containing varying concentrations of auranofin at 37 °C for 48 h. At the end of the growth period, we established each strain’s MIC of auranofin by measuring the optical density of each culture at 600 nm (OD_600_). With this method, we first determined the auranofin sensitivity of wild-type strains R20291 (MIC = 2 µg/mL) and JIR8094 (MIC = 8 µg/mL) (Figs. 1A and 2A). The standard-of-care CDI therapeutics, fidaxomicin and vancomycin, were also included as positive controls for the assay. Accordingly, R20291 was inhibited by 0.125 µg/mL fidaxomicin and 1 µg/mL vancomycin while JIR8094 was inhibited by 0.016 µg/mL fidaxomicin and 4 µg/mL vancomycin (Supplementary Fig. S1).

Based on our laboratory’s previous work, it was proposed that auranofin inhibits the growth of *C. difficile* by forming a complex with Se, thereby depleting the amount of bioavailable Se for trafficking and eventual incorporation into selenoproteins^19^. Thus, if auranofin’s activity arises from the inhibition of selenoprotein biosynthesis, a strain lacking selenoproteins (i.e., a *selD* mutant) would theoretically be resistant to the compound and harbor a significantly elevated MIC compared to wild type. Despite this assumption, we surprisingly found that the MICs for R20291 (Fig. 1A), KNM6 (Δ*selD*) (Fig. 1B), and KNM9 (Δ*selD*::*selD*^+^) (Fig. 1C) were all equally 2 µg/mL auranofin, suggesting that the compound’s activity does not stem from targeting selenoproteins. To determine if this phenomenon was strain dependent, we repeated the assay with JIR8094 and LB-CD7 (*selD*::*ermB*) and likewise saw no increase in the MIC. However, while the wild-type strain JIR8094 exhibited an MIC of 8 µg/mL (Fig. 2A), the *selD*::*ermB* strain was actually more susceptible to auranofin as it failed to grow at 4 µg/mL (Fig. 2B). This slight increase in sensitivity was surprising as it seemed to suggest a complex relationship between auranofin’s antimicrobial activity and the selenoproteins in JIR8094. Out of curiosity, we evaluated two Prd mutants—LB-CD4 (*prdB*::*ermB*) and LB-CD8 (*prdR*::*ermB*)—and one Grd mutant—LB-CD12 (*grdA*::*ermB*)—using the same assay in order to determine which reductase plays a greater role in this phenomenon, if any. Interestingly, we discovered that all three mutants exhibited the same MIC of 4 µg/mL as the *selD*::*ermB* strain (Fig. 2C,D,E). While these data seem to suggest that a mutation in either of these selenoproteins renders *C. difficile* JIR8094 more sensitive to auranofin, a simple two-fold difference in MIC is likely not enough evidence for this. Regardless, these data clearly show that auranofin inhibits the growth of *C. difficile* in the absence of selenoproteins.

### Selenite supplementation neutralizes auranofin’s activity against *C. difficile* even in the absence of selenoproteins

**Figure 3:**
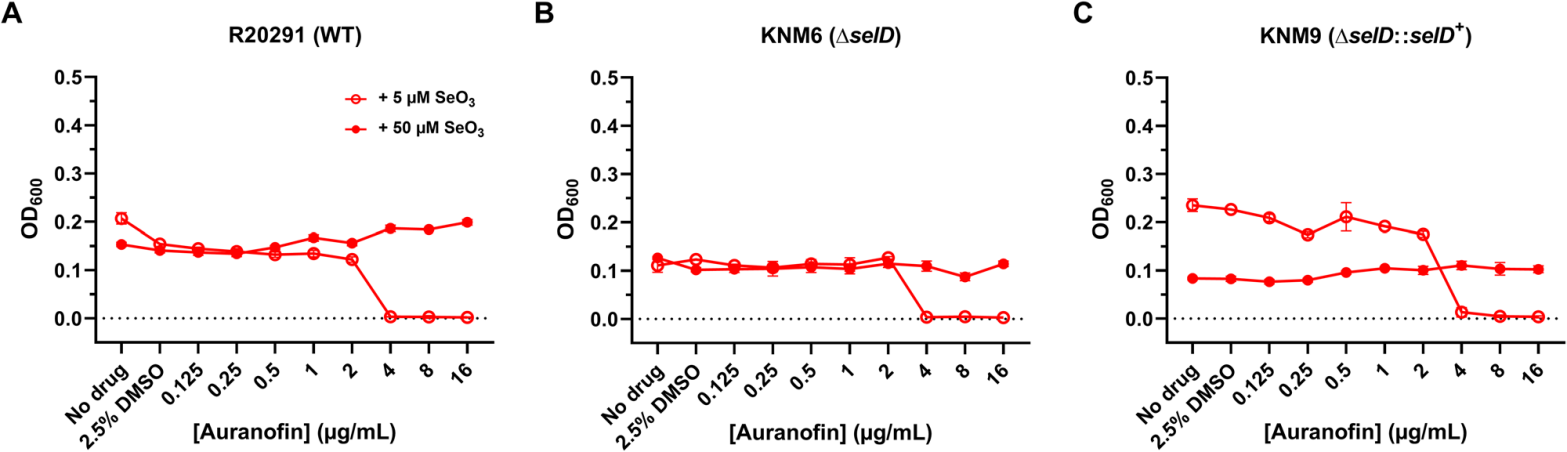
Selenite supplementation decreases auranofin sensitivity even in the absence of selenoproteins. *C. difficile* strains (**A**) R20291, (**B**) KNM6, and (**C**) KNM9 were grown in selenite-supplemented BHIS broth augmented with 2.5% DMSO and varying concentrations of auranofin at 37 °C for 48 h. Sodium selenite was added to give a final concentration of 5 µM (red open circle) or 50 µM (red filled circle). The OD_600_ of each culture was recorded at 48 h. The experiment was performed twice. Data points represent the means of triplicate cultures while error bars represent standard deviations.

**Figure 4:**
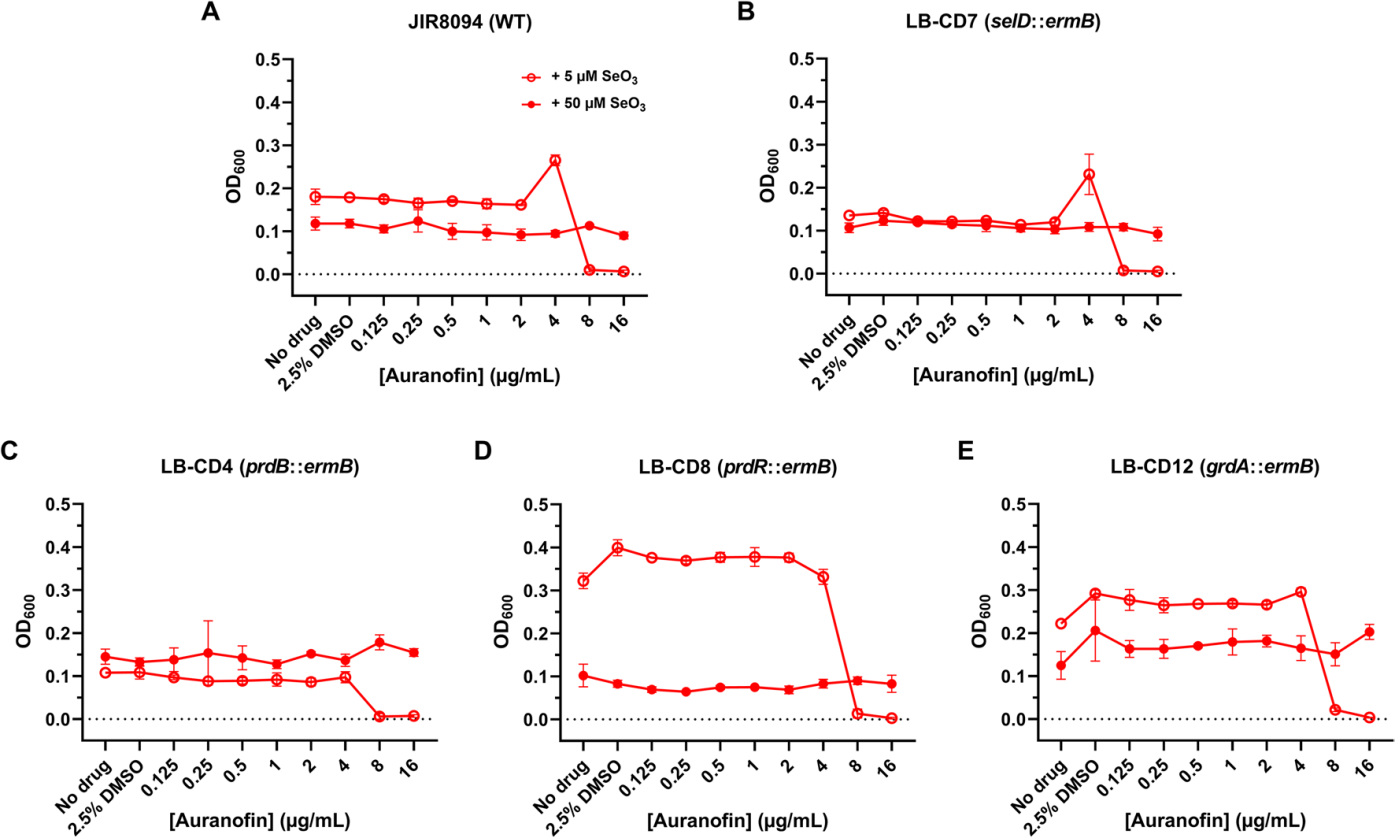
Selenite supplementation decreases auranofin sensitivity in a manner independent of selenophosphate synthetase, proline reductase, or glycine reductase. *C. difficile* strains (**A**) JIR8094, (**B**) LB-CD7, (**C**) LB-CD4, (**D**) LB-CD8, and (**E**) LB-CD12 were grown in selenite-supplemented BHIS broth augmented with 2.5% DMSO and varying concentrations of auranofin at 37 °C for 48 h. Sodium selenite was added to give a final concentration of 5 µM (red open circle) or 50 µM (red filled circle). The OD_600_ of each culture was recorded at 48 h. The experiment was performed twice. Data points represent the means of triplicate cultures while error bars represent standard deviations.

We previously demonstrated that supplementing the culture medium with Se (either as sodium selenite or L-selenocysteine) exhibits a protective effect against auranofin, which we had interpreted as excess Se overcoming the apparent nutritional deficiency caused by the formation of Au-Se adducts^19^. Since auranofin still inhibits the growth of *selD* mutants as well as wild-type strains, we wanted to determine if selenite supplementation would still influence auranofin’s antibacterial activity. When we repeated the previous assay using BHIS broth augmented with 5 µM selenite, we surprisingly observed a two-fold increase in the MICs of all strains (with the exception of JIR8094) (Figs. 3 and 4), suggesting that excess selenite dampened auranofin’s activity regardless of whether selenoproteins were present. To verify if this response could be exacerbated at higher doses, we repeated the same assay with 50 µM selenite. Under these conditions, all strains grew regardless of the auranofin concentration (Figs. 3 and 4). These results clearly demonstrate that selenite’s protective effect against auranofin cannot be explained as simply overcoming a Se deficiency imposed by the compound. While this phenomenon could potentially be interpreted as chemical inactivation by Se, it must be noted that Thangamani et al. reported no selenite-dependent neutralization of auranofin’s activity against methicillin-resistant *Staphylococcus aureus*^38^, suggesting that there are different species-specific mechanisms at play. Finally, given the fact that selenite exhibits varying toxicity to some bacteria^39,40^, we wanted to determine if this was potentially acting as a confounding variable in our experiments. When we cultured our strains in BHIS broth containing varying selenite concentrations, we subsequently observed no difference in growth yields even up to 100 µM (Supplementary Figs. S2 and S3). This result correlates with a publication that reports a staggering MIC of 27 mM sodium selenite against two *C. difficile* isolates^41^.

## Discussion

In this work, we unexpectedly discovered that auranofin inhibits the growth of *C. difficile* mutants lacking selenoproteins. This result was perplexing as we originally thought that auranofin’s antimicrobial activity against *C. difficile* was mainly due to the inhibition of Se metabolism^19^. Our idea had been supported by several lines of evidence: (i) auranofin prevented the uptake of ^75^Se and its incorporation into selenoproteins in both *C. difficile* and anaerobically grown *Escherichia coli*; (ii) the anaerobic growth yield of an *E. coli* Δ*selD* mutant was unaffected by auranofin compared to wild type; and (iii) auranofin exhibited little to no activity against *Clostridium perfringens* and *Clostridium tetani* (i.e., clostridia that lack selenoproteins)^19^. Additionally, we had found that the oral pathogen *Treponema denticola*—an organism with a strict Se requirement for growth^42^—was also susceptible to auranofin, as the compound likewise prevented the uptake and incorporation of ^75^Se into its selenoproteins^43^. Consistent with our initial observations of auranofin’s activity against *C. difficile*^19^, the compound’s growth inhibition of *T. denticola* could be attenuated by supplementation with either sodium selenite or L-selenocysteine^43^. Clearly, our idea of targeting Se metabolism in *C. difficile* was predicated on the assumption that the pathogen required Se for growth in the same manner as *T. denticola*, when in reality, genetic techniques have revealed that selenoproteins are actually not essential to *C. difficile*^28^. Thus, when dealing with organisms that carry dispensable selenoproteins (e.g., *E. coli* and *C. difficile*), Se metabolism becomes a poor candidate for a drug target. Moreover, it is obvious that auranofin’s effects in these bacteria are far more complex than initially assumed; for example, it is unknown why an *E. coli selD* mutant gains slight resistance to auranofin while a *C. difficile selD* mutant exhibits no appreciable change in sensitivity. Further research should focus on fully characterizing the compound’s multiple modes of action in order to truly understand their effects in different pathogens.

As of now, auranofin’s mechanism of action against *C. difficile* is unknown, but a promising candidate may exist within the thioredoxin (Trx) system, which utilizes disulfide reductase activity to protect cytosolic components against oxidative stress and maintain thiol redox homeostasis^44^. The Trx system is comprised of Trx, Trx reductase (TrxR), and NADPH^44^. Trx reduces aberrant disulfides in the cell using a thiol-disulfide exchange mechanism that inevitably causes itself to be oxidized; TrxR utilizes electrons from NADPH to reduce Trx, allowing it to resume its surveillance of the cytosol for more oxidized substrates^44^. Interestingly, auranofin is known to be a selective inhibitor of TrxR in mammalian cells and parasites^45–47^. Likewise, auranofin has been shown to inhibit bacterial TrxR in some clinical pathogens such as *Mycobacterium tuberculosis*, *S. aureus*, and *Helicobacter pylori*^48–50^. Harbut et al.^48^ even proposed that auranofin’s poor activity against several Gram-negative bacteria is actually due to the presence of the glutathione system, which can provide compensatory disulfide reductase activity in the event of a compromised Trx system. Thus, in bacteria lacking glutathione (i.e., most Gram-positives), auranofin-dependent inhibition of TrxR is expected to be lethal. It is therefore tempting to believe that auranofin could be exhibiting a similar mechanism in *C. difficile* due to two important observations: (i) a *trxR* gene exists within the *grd* operon^25^, and (ii) the cysteine-to-glutathione biosynthesis pathway is reportedly absent from the genome^51^. Alternatively, Thangamani et al. claimed that auranofin likely possesses multiple modes of action, as the compound was able to inhibit several biosynthetic pathways in *S. aureus* (e.g., DNA, protein, and cell wall syntheses)^38^. Moreover, the authors suggest that auranofin’s weak activity against Gram-negatives may instead be due to the presence of the outer membrane and efflux pumps, rather than the redundant activity of the glutathione system^38^. Specifically, they showed that several Gram-negative pathogens were only susceptible to auranofin when the permeabilizing agent polymyxin B nonapeptide was present; moreover, an *E. coli* double mutant lacking both TrxR (*trxB*) and glutathione reductase (*gor*) did not differ in auranofin sensitivity compared to wild type^38^. Overall, these data imply that inhibition of TrxR—akin to inhibition of selenoprotein synthesis in *C. difficile*—may not be the only mechanism that this compound utilizes against bacteria. A classic technique to determine the mechanism of action of an antimicrobial involves the careful isolation of spontaneous drug-resistant mutants in vitro; however, numerous groups have clearly reported an inability to generate spontaneous auranofin-resistant mutants of several bacterial species using this method^38,48,50,52–54^. Likewise, our attempts to isolate spontaneous auranofin-resistant *C. difficile* mutants were met with failure, which further supports the idea of auranofin possessing multiple modes of action.

## Materials and methods

### Bacterial strains and growth maintenance

All *C. difficile* strains used in this study are listed in Table 1. Growth experiments were performed in a Coy anaerobic chamber under an atmosphere of ~1.0% H_2_, 5% CO_2_, and >90% N_2_. Strains were routinely maintained on BHIS agar (37 g/L brain heart infusion, 5 g/L yeast extract, 0.1% L-cysteine). When indicated, overnight cultures were prepared by inoculating 5 mL BHIS broth with single colonies of the appropriate strains followed by 16–24 h of incubation at 37 °C.

### Broth microdilution assay

MICs were determined using a modified broth microdilution assay as per the CLSI M11^33^. Briefly, auranofin was dissolved in 100% dimethyl sulfoxide (DMSO) and subsequently diluted to achieve working stocks at 20× concentration in 50% DMSO. Similarly, fidaxomicin was dissolved in 100% DMSO while vancomycin hydrochloride was dissolved in deionized water. Diluted test compounds (5 μL) were mixed with 95 μL BHIS broth in triplicate in 96-well plate format. Plates were reduced overnight in the anaerobic chamber. On the day of experimentation, overnight cultures of all strains were diluted to match a 0.5 McFarland standard and were subsequently diluted again 15-fold using pre-reduced 0.85% NaCl. Diluted cells (10 μL) were used to inoculate 100 μL triplicate drug-broth mixtures in pre-reduced 96-well plates, which were then incubated in half-sealed plastic bags at 37 °C for 48 h. After incubation, the OD_600_ was recorded for all cultures and normalized by subtracting the mean OD_600_ of triplicate uninoculated BHIS broth controls (blank correction). MICs were scored as the lowest concentration of compound that resulted in non-turbid cultures as compared to uninoculated controls after 48 h. For all selenite supplementation experiments, the assay described above was performed with BHIS broth augmented with either 5 or 50 μM sodium selenite in triplicate. Blank corrections were done relative to the appropriate selenite concentration, as selenite imparts a slight red color to the medium. All experiments were performed twice. Statistical analysis (mean OD_600_ ± s.d.) was performed using GraphPad Prism 9.

### Selenite sensitivity assay

Overnight cultures of all strains were diluted 100-fold in BHIS broth augmented with 0, 5, 10, 25, 50, or 100 µM sodium selenite in triplicate. Diluted cultures were grown for 24 h at 37 °C. The OD_600_ was recorded for all cultures at the end of the growth period. Blank corrections were done relative to the appropriate selenite concentration as described above. The experiment was performed twice. Statistical analysis (mean OD_600_ ± s.d.) was performed using GraphPad Prism 9.

### Supplementary Information

Supplementary Figures.

## Data Availability

The data are available from the corresponding author upon reasonable request.
