# Supplementary material for: Inhibition of selenoprotein synthesis is not the mechanism by which auranofin inhibits growth of Clostridioides difficile
Source: Sci Rep. 2023 Sep 7;13:14733. doi: 10.1038/s41598-023-36796-9 (PMC10484987; doi:10.1038/s41598-023-36796-9)
Supplement: Supplementary file 1 — Supplementary Figures. [file 41598_2023_36796_MOESM1_ESM.docx]

**Inhibition of selenoprotein synthesis is not the mechanism by which auranofin inhibits growth of *Clostridioides difficile***

Michael A. Johnstone, Matthew A. Holman, and William T. Self*

Burnett School of Biomedical Sciences, College of Medicine, University of Central Florida

* Corresponding author: william.self@ucf.edu

**Supplementary Figure S1.** **Fidaxomicin and vancomycin activity against R20291 and JIR8094.**

**Supplementary Figure S2.** **The R20291 strains are not sensitive to selenite up to 100 µM.**

**Supplementary Figure S3. The JIR8094 strains are not sensitive to selenite up to 100 µM.**


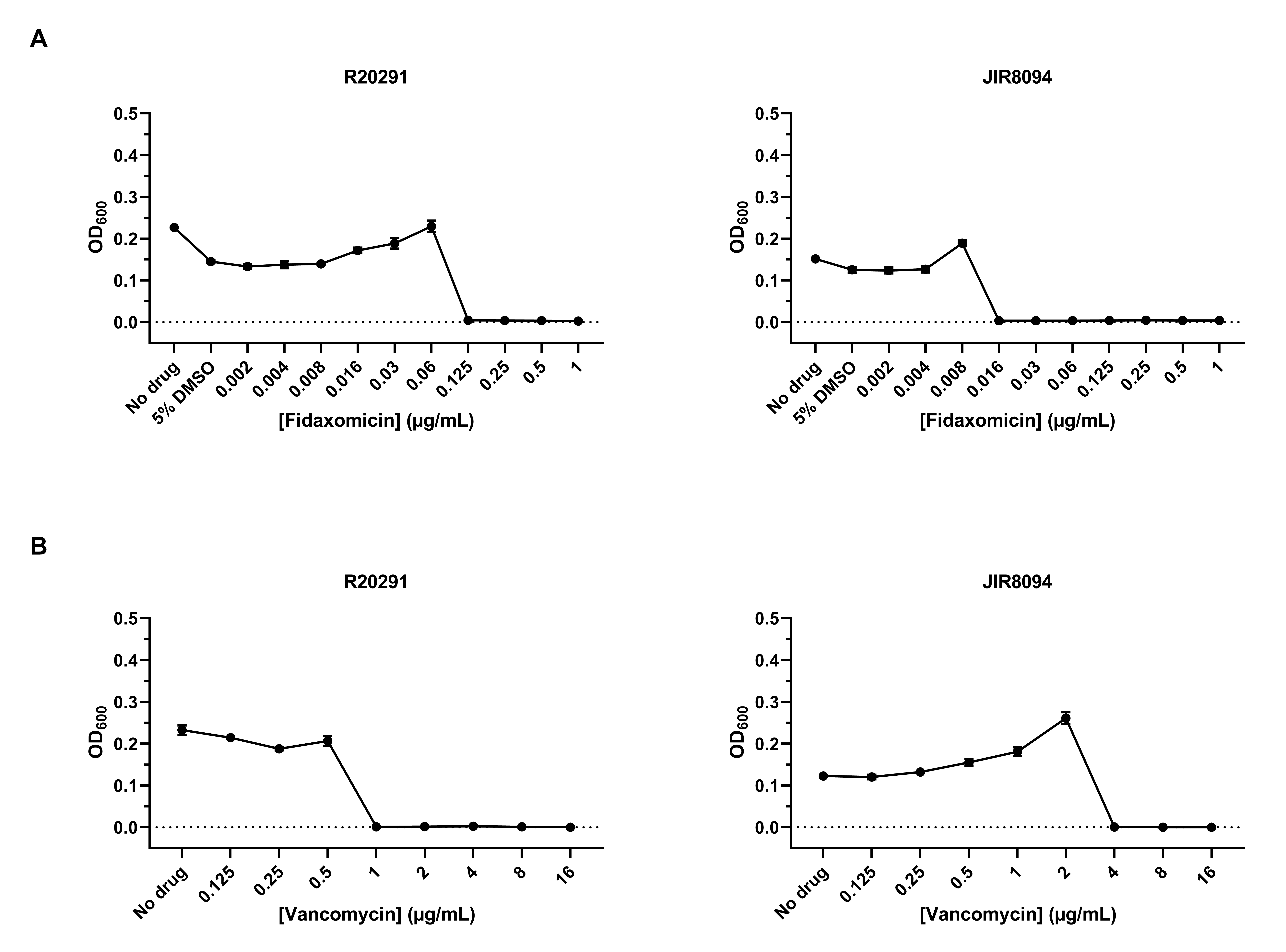


**Supplementary Figure S1.** **Fidaxomicin and vancomycin activity against R20291 and JIR8094.** C. difficile strains R20291 and JIR8094 were grown in BHIS broth with varying concentrations of (A) fidaxomicin and (B) vancomycin at 37 °C for 48 h. The OD_600_ of each culture was recorded at 48 h. The experiment was performed twice. The vehicle control for fidaxomicin was 5% DMSO. A vehicle control was not included for vancomycin as it was dissolved in dH_2_O. Data points represent the means of triplicate cultures while error bars represent standard deviations.


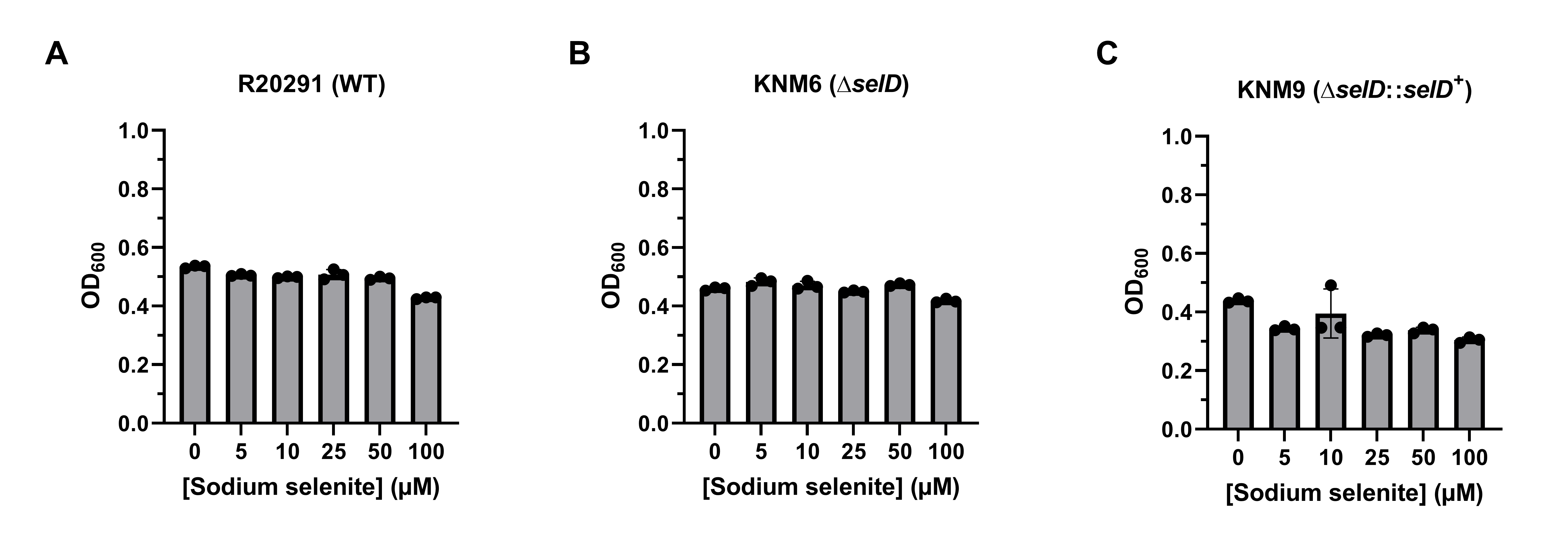


**Supplementary Figure S2.** **The R20291 strains are not sensitive to selenite up to 100 µM.** C. difficile strains (A) R20291, (B) KNM6, and (C) KNM9 were grown in BHIS broth supplemented with 0, 5, 10, 25, 50, or 100 µM sodium selenite at 37 °C for 24 h. The OD_600_ of each culture was recorded at 24 h. The experiment was performed twice. Data points represent the means of triplicate cultures while error bars represent standard deviations.


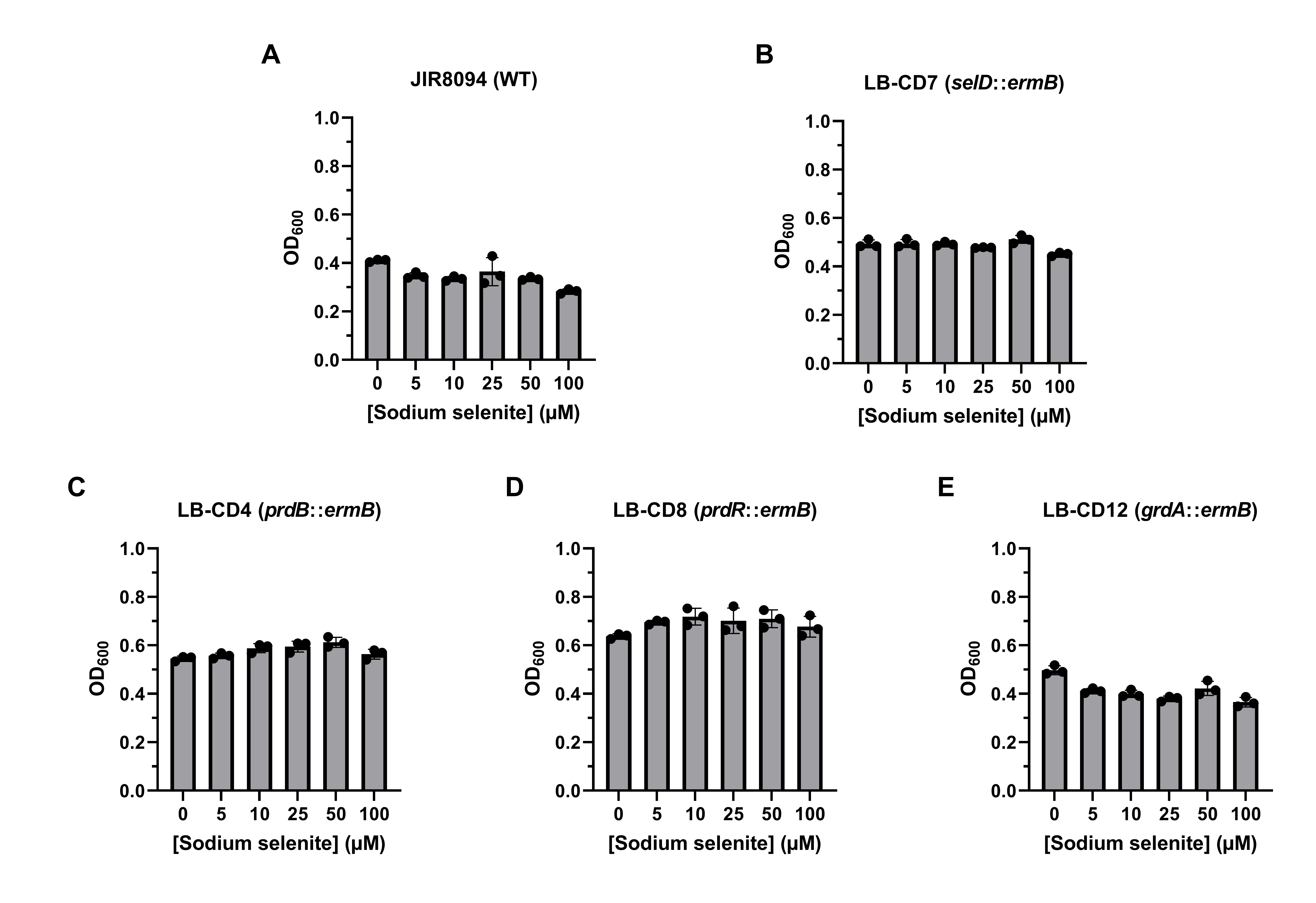


**Supplementary Figure S3.** **The JIR8094 strains are not sensitive to selenite up to 100 µM.** C. difficile strains (A) JIR8094, (B) LB-CD7, (C) LB-CD4, (D) LB-CD8, and (E) LB-CD12 were grown in BHIS broth supplemented with 0, 5, 10, 25, 50, or 100 µM sodium selenite at 37 °C for 24 h. The OD_600_ of each culture was recorded at 24 h. The experiment was performed twice. Data points represent the means of triplicate cultures while error bars represent standard deviations.
